# Altered cutaneous reflexes to non-noxious stimuli in the triceps surae of people with chronic incomplete spinal cord injury

**DOI:** 10.1152/jn.00266.2022

**Published:** 2023-02-01

**Authors:** Alan M. Phipps, Aiko K. Thompson

**Affiliations:** Department of Health Sciences and Research, College of Health Professions, Medical University of South Carolina, Charleston, South Carolina, United States

**Keywords:** H-reflex, hyperreflexia, locomotion, spinal reflexes, task-dependent modulation

## Abstract

Following spinal cord injury (SCI), task-dependent modulation of spinal reflexes is often impaired. To gain insight into the state of the spinal interneuronal pathways following injury, we studied the amplitude modulation of triceps surae cutaneous reflexes to non-noxious stimuli during standing and early-to-mid stance phase of walking in participants with and without chronic incomplete SCI. Reflex eliciting nerve stimulation was delivered to the superficial peroneal, sural, and distal tibial nerves about the ankle. Reflexes were analyzed in the short (SLR, 50–80 ms poststimulation onset) and the medium (MLR, 80–120 ms) latency response windows. Furthermore, the relation between cutaneous and H-reflexes was also examined during standing. In participants without injuries the soleus SLR was modulated task dependently with nerve specificity, and the soleus and medial gastrocnemius MLRs were modulated task dependently. In contrast, participants with SCI, no task-dependent or nerve-specific modulation of triceps cutaneous reflexes was observed. The triceps surae cutaneous and H-reflexes were not correlated in either group (*r* = 0.01–0.37). The presence of cutaneous reflexes but the absence of significant amplitude modulation may suggest impaired function of spinal interneuronal pathways in this population. The lack of correlation between the cutaneous and H-reflexes may suggest that interneurons that are involved in H-reflex modulation and cutaneous reflex modulation do not receive common input, or the impact of the common input is outweighed by other input. Present findings highlight the importance of examining multiple spinal reflexes to better understanding spinal interneuronal pathways that affect motor control in people after SCI.

**NEW & NOTEWORTHY** This study examined modulation of the triceps surae cutaneous reflexes during standing and walking and the relationship between cutaneous and H-reflexes in people with chronic incomplete spinal cord injury (SCI). In people with SCI, the normal task-dependent, nerve-specific modulation of triceps cutaneous reflexes was missing. Cutaneous and H-reflexes were not correlated. Together with other spinal reflexes, cutaneous reflexes may serve as important biomarkers for the state of spinal interneuronal pathways.

## Graphical abstract

**Figure:**
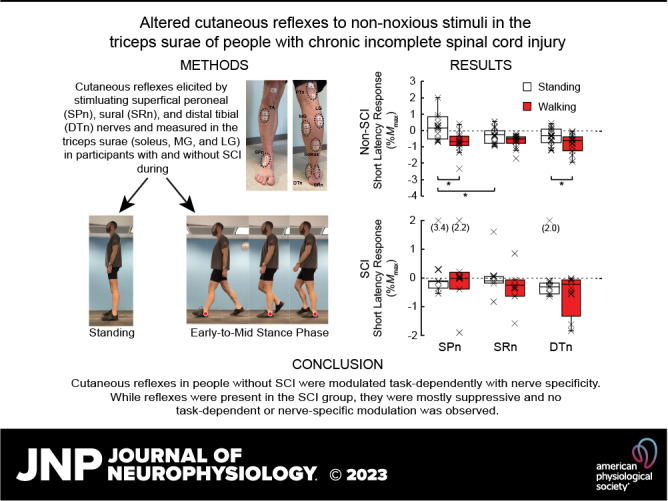

## INTRODUCTION

Normally, spinal reflexes are task-dependently modulated so that they can function appropriately in each motor task being performed (1). For example, the soleus H-reflex and reciprocal inhibition become progressively smaller from standing to walking to running (2–4). Cutaneous reflexes to non-noxious stimulation of Aβ afferents (5) increase in amplitude from standing to running (6). Among different spinal reflexes, cutaneous reflexes are unique in how they modulate; depending on the task, phase of the movement cycle, latency component, and nerve stimulated, the cutaneous reflex response can be excitatory or inhibitory (6–13). The fact that cutaneous reflexes can be inhibitory or excitatory may reflect the powerful modulatory influence that cutaneous input exerts over multiple spinal segments via interneuronal pathways (14). Previous studies suggest that cutaneous reflexes to non-noxious cutaneous stimuli help to shape multi-joint movements/behaviors, e.g., through “stumbling corrective response” across multiple muscles (1, 13, 15) and (potential) obstacle avoidance during locomotion (8–11, 16). Overall, the presence of cutaneous reflexes and the extent of their modulation (e.g., task- or phase-dependent modulation) indicate the availability of those reflex pathways in different tasks, phases, and circumstances, and reflect how cutaneous input is processed and used by the central nervous system (CNS).

After spinal cord injury (SCI) disrupts supraspinal connections, the activity and excitability of spinal reflex pathways change (17–21), reflecting altered spinal somatosensory processing in those individuals. Function and behavior of excitatory reflexes arising from muscle spindle afferents become abnormal (17, 18, 20–25) along with changes in reciprocal inhibition (17–19, 26–28), Ib inhibition (29), recurrent inhibition (30), and spinal motoneurons and interneurons (31–34). Of multiple spinal reflexes that play key roles in normal and impaired motor control, cutaneous reflexes are among the least studied reflexes in people after SCI. Within the limited available literature, Jones and Yang (35) demonstrated the distinctly different patterns of cutaneous reflex modulation in the ankle dorsiflexor tibialis anterior (TA) and the plantarflexor soleus during locomotion in people with chronic incomplete SCI. During the late swing phase, the TA response to distal tibial nerve (DTn) stimulation is usually inhibitory in noninjured individuals, but it was found excitatory in most participants with SCI. The soleus response to DTn stimulation was more exaggerated in participants with SCI than in noninjured individuals, both during the stance and swing phases. Knikou et al. (36) stimulated the sural nerve (SRn) during stepping and found short (SLR) and medium latency responses (MLR) only in 3 of 9 individuals after SCI. After these studies (35, 36), several key questions regarding cutaneous reflexes in individuals with SCI remain. For example, in people with chronic incomplete SCI, are cutaneous reflexes modulated task dependently (e.g., standing vs. walking)? Do people after SCI show nerve (i.e., represented skin area) specific responses and/or modulation patterns? Are cutaneous reflex measures related to other spinal reflex measures (e.g., H-reflex amplitude)? Answers to these questions would help to determine the utility of cutaneous reflexes in examining the functional state of spinal cord pathways in people with chronic incomplete SCI.

As the first step in characterizing cutaneous reflexes and their modulation after SCI, this study examined the amplitudes and patterns of triceps surae cutaneous reflexes to non-noxious stimuli during standing and early-to-mid stance phase of walking, in both of which these muscles are active, often to a similar extent. Specifically, we measured cutaneous reflexes to the superficial peroneal (SPn, innervating the foot dorsum), sural (SRn, lateral aspect of the foot), and distal tibial nerve (DTn, medial aspect of foot extending toward the plantar surface of digits one through three) stimulation in the triceps surae of ambulatory individuals with chronic SCI and individuals with no known neurological conditions. Cutaneous reflexes were examined in two latency components: short latency response (SLR) and medium latency response (MLR). SLR is typically observed 50–80 ms poststimulus onset in the lower leg muscles and is mediated by spinal mechanisms (37). MLR is typically observed 80–120 ms poststimulus and also spinally mediated, but likely influenced by supraspinal input and known to exhibit reflex reversals during walking (e.g., inhibition during swing-to-stance transition and excitation during swing phase in the TA) (11). Since Jones and Yang (35) and Knikou et al. (36), several studies have examined the modulation of other spinal reflexes by cutaneous input in people after SCI (38–40), but to our knowledge, this is the first study to examine the presence/absence, task-dependent modulation, and nerve specificity of triceps surae cutaneous reflexes in ambulatory individuals with chronic SCI.

## METHODS

### Participants

Eight individuals with chronic incomplete SCI (4 males, 4 females) aged 20–74 yr (56.1 ± 16.5 yr, means ± SD) and 14 individuals with no known neurological conditions (age-matched non-SCI group; 7 males, 7 females) aged 72–21 yr (48.3 ± 15.0 yr) participated in this study. Profiles of individuals with SCI are summarized in Table 1. Before testing, all participants provided written informed consent that was approved by the Institutional Review Board of the Medical University of South Carolina.

**Table 1.** Participant characteristics

ID	Age, yr	Sex	Injury Level	Time Since SCI, yr	Cause of Injury	Walking Speed, m/s	Baclofen	*H*_max_ (%*M*_max_)		
								Soleus	MG	LG
1	62	F	C4-C5	13	T	0.4	N	89	32	31
2	60	M	C5-C6	15	T	0.4	Y	24	18	27
3	74	M	C1-C4	50	T	0.1	Y	35	19	18
4	20	F	C3-C5	4	NT	0.4	N	91	44	42
5	61	F	C5	10	NT	0.5	N	71	38	45
6	66	M	C3-C6	2	NT	0.4	N	75	52	63
7†	60	M	C3-C7	8	T	0.1	Y	76	24	35
8	46	F	T10-T12	1	NT	0.7	N	92	75	66
Non-SCI	48.3 ± 15.0*					1.0 ± 0.2*		45 ± 24*	12 ± 10*	11 ± 7*

F, female; LG, lateral gastrocnemius; M, male; MG, medial gastrocnemius; NT, nontraumatic; SCI, spinal cord injury; T, trauma.

† Subject walked overground. Walking speed is the speed used during locomotor reflex measurements; *means ± SD.

The inclusion criteria for participants with SCI were *1*) neurologically stable (>6 mo after lesion), *2*) medically stable (i.e., no changes to medication for at least 3 mo), and *3*) ability to ambulate with or without an assistive device (except parallel bars) at least 10 m. Note that chronic stable use of antispasticity medication such as baclofen, diazepam, or tizanidine was accepted. All participants with SCI exhibited clinical signs of spasticity (i.e., increased muscle tone, score ≥1 on Modified Ashworth scale) at least unilaterally. Exclusion criteria were *1*) lower motor neuron injury, *2*) known cardiac conditions, *3*) medically unstable conditions, *4*) cognitive impairment, *5*) uncontrolled peripheral neuropathy, *6*) extensive use of functional electrical stimulation (e.g., foot-drop stimulator) on a daily basis, and *7*) complete lack of cutaneous sensation around the foot.

Participants in the age-matched non-SCI group were free from *1*) known neurological conditions and *2*) lower limb orthopedic injuries within the past year.

In each participant with SCI, the more affected leg, which was defined as the one with more severe spasticity and confirmed by the research occupational therapist (BD), was studied. In participants of the non-SCI group, the left leg was studied.

Note that the SCI group and non-SCI group were different in walking speed used for reflex measurements (0.4 ± 0.2 m/s [means ± SD] for the SCI group vs. 1.0 ± 0.2 m/s for the non-SCI group, *P* < 0.001).

### General Procedures

**Figure 1.**
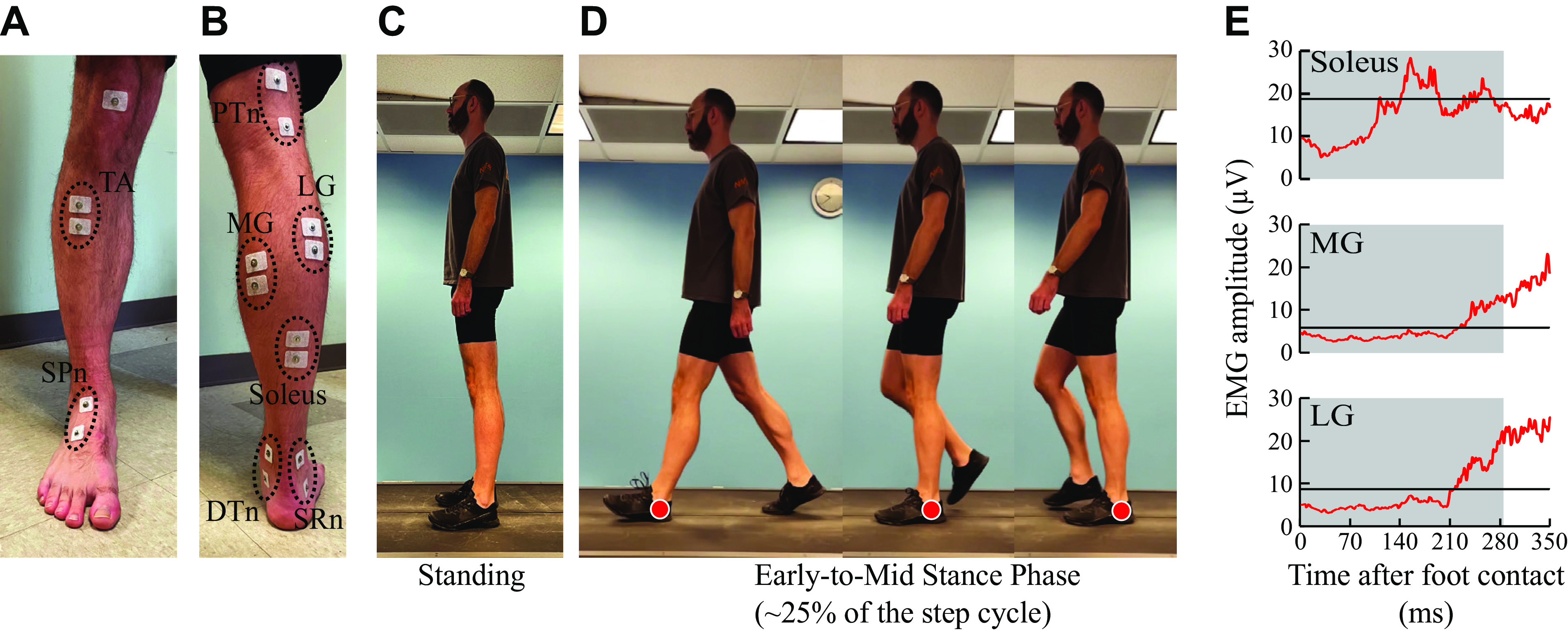
Experimental setup. *A* and *B*: locations of electromyography (EMG) recording and nerve stimulating electrodes are shown. DTn, distal tibial nerve; LG, lateral gastrocnemius; MG, medial gastrocnemius; PTn, posterior tibial nerve; SPn, superficial peroneal nerve; SRn, sural nerve; TA, tibialis anterior. *C* and *D*: the participant’s posture during standing (*C*) and the first ∼25% of the step cycle of walking (*D*). The red markers in *D* indicate the left leg that is the studied leg in this example. *E*: typical EMG activity during the early-to-mid stance phase of walking is shown in red. One hundred ten nonstimulated steps were averaged together for the participant of this example [non-spinal cord injury (SCI)]. Black lines indicate background EMG levels during standing. The gray shaded areas highlight the early-to-mid stance phase (i.e., the first ∼25% of the step cycle) during which cutaneous reflexes were elicited.

At the beginning of the study, electromyography (EMG) recording and stimulating electrodes for cutaneous (SPn, SRn, and DTn) and posterior tibial nerve (PTn) were placed over the leg (Fig. 1, *A* and *B*). An H-reflex-M-wave recruitment curve was obtained for the triceps surae while participants stood and maintained a preset level of soleus (i.e., close to the natural standing level) and tibialis anterior (TA) background EMG activity (see *EMG and Nerve Stimulation*) (Fig. 1*C*). Then, cutaneous reflexes were elicited by SPn, SRn, and DTn stimulation separately, while the participant stood and maintained the preset level of soleus and TA EMG activity (Fig. 1*C*), or during early-to-mid stance phase of walking at his/her comfortable speed (Fig. 1*D*).

### EMG and Nerve Stimulation

EMG signals were recorded continuously from the triceps surae [soleus, medial gastrocnemius (MG), and lateral gastrocnemius (LG)] and TA, ipsilateral to the stimulation site, using pairs of self-adhesive surface Ag-AgCl electrodes (2.2 × 3.3 cm, Vermed/Nissha Medical Technologies, Buffalo, NY) (Fig. 1, *A* and *B*) with their centers ∼3 cm apart. TA, MG, and LG electrodes were placed over the muscle belly, and soleus electrodes were placed below the gastrocnemius and in line with the Achilles tendon. EMG signals were amplified, bandpass filtered at 10–1,000 Hz, sampled at 4,000 Hz, and stored.

To elicit the H-reflex and M-wave in the triceps surae, the PTn was stimulated in the popliteal fossa, using Ag-AgCl electrodes (2.2 × 2.2 cm for the cathode and 2.2 × 3.3 cm for the anode, Vermed). Stimulating electrodes were placed to reduce H-reflex threshold, maximize the M-wave size, and avoid stimulation of other nerves. Single square pulses (1–2 ms in pulse-width) were delivered through a Digitimer DS7A constant current unit (Digitimer Limited, Letchworth Garden City, UK) when the participant had maintained the natural standing level of soleus (corresponding to 10–12 µV in the SCI group and 13–18 µV in the non-SCI group) and TA (<6.0 µV in both groups, essentially equivalent to no activity) EMG activity for at least 2 s. The minimum interstimulus interval was 5 s. The H-reflex-M-wave recruitment curves were obtained by gradually increasing the PTn stimulus intensity from below the soleus H-reflex threshold to the maximum H-reflex (*H*_max_) to beyond the level required to elicit the maximum M-wave (*M*_max_) (41–43). Two to four responses were averaged at each intensity (∼40 trials in total) to obtain the recruitment curve.

Cutaneous reflexes were elicited by stimulating either the SPn, SRn, or DTn using two 2.2 × 2.2 cm electrodes (Vermed). For SPn stimulation, the anode was positioned near the anterior portion of the ankle and cathode on the foot dorsum. The SRn was stimulated around the posterior aspect of the lateral malleolus while the DTn was stimulated slightly posterior and/or distal to the medial malleolus (Fig. 1, *A* and *B*). Cutaneous nerve stimulation was delivered as train of five 1.0-ms pulses at 200 Hz through a Grass S48 stimulator (Natus Neurology Grass, Warwick, RI) and a Digitimer DS7A constant current unit (Digitimer Limited). For each nerve stimulated, stimulus electrode locations were optimized to generate the strongest cutaneous sensation over the largest skin area at a given stimulus current. Following optimization, perceptual (PT) and radiating thresholds (RT) were determined in standing. PT was defined as stimulus intensity in which the first discernible sensation could be recognized. RT was determined by increasing the stimulus current until a strong radiating paresthesia in the innervation area was indicated by the participant.

To examine cutaneous reflexes to non-noxious stimuli, cutaneous nerve stimulation at 2 × RT was delivered during standing and early-to-mid stance phase of walking, similarly to the previous studies (7, 8, 16, 44). In participants who perceived 2 × RT stimulation as painful, the stimulus intensity was reduced to a non-noxious tolerable level. For each nerve stimulation condition, 20 cutaneous reflex trials were administered at the fixed intensity (i.e., 2 × RT or less) while the participant maintained the standing posture and the preset level of soleus and TA background EMG activity. Then, at a self-selected comfortable speed, all but one participant walked on an instrumented treadmill (Bertec Corp., Columbus, OH) with a safety harness on while cutaneous nerve stimulation was delivered. The stimulus trigger timing was generally related to the time of foot contact, and the timing of stimulation was pseudo-randomly varied around foot contact (i.e., from just before foot contact through mid-stance) so that the SLR and MLR reflexes (with latencies of 50–80 ms poststimulus onset for SLR and 80–120 ms for MLR, typically) would be measured in early-to-mid stance phase (i.e., 0–25% of the step cycle) of walking (Fig. 1*D*). Any reflexes that occurred outside the early-to-mid stance phase were excluded from the study’s data pool during postexperiment offline data analysis. Each participant walked on the treadmill for ≈5 min (200–300 steps) per nerve stimulation condition. In one participant with SCI, all locomotor reflex measurements were performed overground since the laboratory design around the treadmill could not accommodate this participant’s limited gait function. The interstimulus-train interval was set such that at least one full unstimulated step was taken between stimulus trains.

To detect foot contact, ground reaction force signal from the instrumented treadmill’s force plates or the signal from foot-switch cells inserted between the participant’s shoe and foot were used. The step cycle was defined as the cycle between two consecutive foot contacts (i.e., from the beginning of stance to the end of swing phase). In this study, we analyzed the data from the first 25% of the step cycle (i.e., early-to-mid stance phase), as the posture and triceps EMG activity during this phase of step cycle are somewhat similar to those during standing (see Fig. 1).

### Data Analysis

#### H_max_ and M_max_.

In each of the triceps surae for each participant, *H*_max_ and *M*_max_ sizes were calculated in the full-wave rectified EMG signal as the difference between the background (i.e., prestimulus) EMG and the mean amplitude over the response (i.e., *H* or *M*) window, which was determined visually with the following criteria. For the *M*_max_, the onset was defined as the first time after PTn stimulation when the EMG amplitude exceeded ≈20% of the peak amplitude and the end was defined as the time when the EMG amplitude subsided below ≈20% of the peak amplitude within the continuum of *M*_max_. For the H-reflex, the onset was defined as the first time after the *M*_max_ window when the EMG amplitude exceeded the background EMG (i.e., mean prestimulus EMG measured over 50 ms before tibial nerve stimulation) and the end was defined as the time after the peak when the EMG amplitude subsided below the background EMG level. Typical *M*_max_ windows were 7–22 ms, 6–20 ms, and 6–20 ms poststimulus for the soleus, MG, and LG, respectively. Typical *H*_max_ windows were 35–48 ms, 34–46 ms, and 34–45 ms poststimulus for the soleus, MG, and LG, respectively (41, 42, 45).

#### Cutaneous reflexes.

All EMG signals were full-wave rectified and low-pass filtered at 100 Hz for analysis. For standing, 20 trials were collected and averaged for each nerve stimulation condition. For walking, responses to stimuli that occurred during the first 25% of the step cycle (i.e., early-to-mid stance phase) were averaged together (*n* = 19–24 in each participant). For each nerve condition, each participant’s SLR and MLR windows were determined individually through visual inspection on the averaged EMG sweeps. When there was no discernable cutaneous reflex response or separating SLR from MLR was difficult, we adopted the latency windows that were used in the previous studies (46–49) (i.e., 50–80 ms poststimulus train onset for SLR and 80–120 ms for MLR) for these analyses. To calculate the reflex amplitudes, the control EMG amplitude was subtracted from the EMG amplitude over a cutaneous reflex (i.e., SLR and MLR) window. That is, for standing, the background EMG averaged over 50 ms of prestimulus period was subtracted from the EMG amplitude over SLR (or MLR) window to calculate the SLR (or MLR) amplitude for each trial. For walking, the averaged EMG from nonstimulated steps (typically >100 steps for each nerve stimulation condition in each of the participants) served as the control EMG (2, 8, 11, 25), and the amplitude of the control EMG from the time in the step cycle corresponding to the timing of SLR (or MLR) was subtracted from the stimulated step’s EMG amplitude over the SLR (or MLR) window. For both standing and walking, those single-trial values were averaged for each participant’s each nerve stimulation condition.

To compare standing and walking conditions, each of the triceps surae cutaneous reflex size was normalized to each participant’s mean rectified *M*_max_ measured during standing for a given muscle.

#### Statistical analysis.

To compare participant characteristics between the two groups, an independent *t* test was used. To confirm the consistency in experimental condition across three nerve stimulation conditions, one-way repeated-measures ANOVA was applied to the background/control EMG for soleus, MG, LG, and TA for each group and task. To assess whether cutaneous reflexes were task-dependently modulated with nerve-specificity, two-way repeated-measures ANOVA [i.e., task (standing/walking) × nerve (SRn/SPn/DTn)] was applied to SLR and MLR separately for each group, and a *t* test with a Bonferroni correction was used as a post hoc. Homogeneity of variances was tested with Mauchly’s test of sphericity for repeated measures. When sphericity could not be assumed, a Greenhouse–Geisser correction was performed. Although the assumption of normality was violated on occasion, a two-way ANOVA was appropriate to test the research question, since there is no nonparametric equivalent, and *F* controls for Type I errors in the presence of skewness, kurtosis, and non-normality (50). Effect sizes [partial eta squared (η^2^)] were calculated for all main effects and interactions and Cohen’s *D* for main effects. Finally, to examine whether cutaneous reflex measures are related to the H-reflex measures, Pearson’s *r* (correlation coefficient) for H- and cutaneous reflexes (both reflexes normalized to *M*_max_) were calculated for each muscle. All statistical analyses were completed using IBM SPSS Version 28 and α level was set at 0.05.

## RESULTS

### Short Latency Response during Standing and Walking

For all studied muscles (i.e., soleus, MG, LG, and TA), background EMG was compared across the three nerve stimulation conditions (one-way repeated-measures ANOVA) in standing or walking (early-to-mid stance phase). For the non-SCI group, MG, LG, and TA background EMG did not significantly differ in standing (*F*_1.123,18.015_ = 0.015, *P* = 0.985, partial η^2^ = 0.001; *F*_1.163,15.124_ = 0.803, *P* = 0.403, partial η^2^ = 0.058; and *F*_2,26_ = 0.162, *P* = 0.851, partial η^2^ = 0.012, respectively) or walking (*F*_1.229,15.977_ = 1.844, *P* = 0.195, partial η^2^ = 0.124; *F*_2,26_ = 0.715, *P* = 0.499, partial η^2^ = 0.052; and *F*_1.235,16.049_ = 1.885, *P* = 0.189, partial η^2^ = 0.127, respectively). Soleus background EMG was not significantly different across nerve conditions during standing (*F*_1.355,17.617_ = 0.517, *P* = 0.536, partial η^2^ = 0.038) but was during walking (*F*_2,26_ = 5.241, *P* = 0.012, partial η^2^ = 0.287). Post hoc revealed that soleus background EMG for the SPn stimulation condition was significantly larger than that for the DTn stimulation condition [29 µV (2.9%*M*_max_) for SPn and 25 µV (2.5%*M*_max_) for DTn, *P* = 0.026]. Although the difference was statistically significant, it is unlikely that such a small magnitude of background EMG difference affected the present assessment of cutaneous reflex modulation. In the SCI group, there was no significant difference across nerve conditions for the soleus, MG, LG, or TA during standing (*F*_2,12_ = 0.683, *P* = 0.524, partial η^2^ = 0.102; *F*_2,10_ = 0.905, *P* = 0.435, partial η^2^ = 0.153; *F*_2,10_ = 1.740, *P* = 0.225, partial η^2^ = 0.258, and *F*_2,12_ = 0.799, *P* = 0.473, partial η^2^ = 0.117, respectively) or walking (*F*_2,12_ = 0.035, *P* = 0.965, partial η^2^ = 0.006; *F*_2,12_ = 0.201, *P* = 0.820, partial η^2^ = 0.032; *F*_2,12_ = 1.757, *P* = 0.214, partial η^2^ = 0.227, and *F*_1.029,6.174_ = 0.065, *P* = 0.831, partial η^2^ = 0.011, respectively). Thus, any differences in cutaneous reflexes between nerve stimulation conditions would be due to nerve-specific modulation. Figure 1*E* shows the soleus, MG, and LG background EMG levels for standing (black lines) and during early-to-mid stance phase of walking (red lines) in one participant without SCI.

Between the SCI and non-SCI groups, there was no significant difference in PT (SPn *P* = 0.589; SRn *P* = 0.900; DTn *P* = 0.445), RT (SPn *P* = 0.233; SRn *P* = 0.263; DTn *P* = 0.328), or stimulus intensity as a multiple of RT (SPn *P* = 0.318; SRn *P* = 0.330; DTn *P* = 0.470). Absolute stimulus current (mA) did not differ significantly between the groups for SRn (13.9 ± 6.0 mA for the SCI group vs. 13.1 ± 4.5 mA for the non-SCI group, *P* = 0.731) and DTn (13.0 ± 5.1 mA for the SCI group vs. 11.7 ± 3 ± 3.8 mA for the non-SCI group, *P* = 0.500) stimulation but did for SPn stimulation (13.4 ± 5.3 mA for the SCI group vs. 18.9 ± 5.5 mA for the non-SCI group, *P* = 0.043).

**Figure 2.**
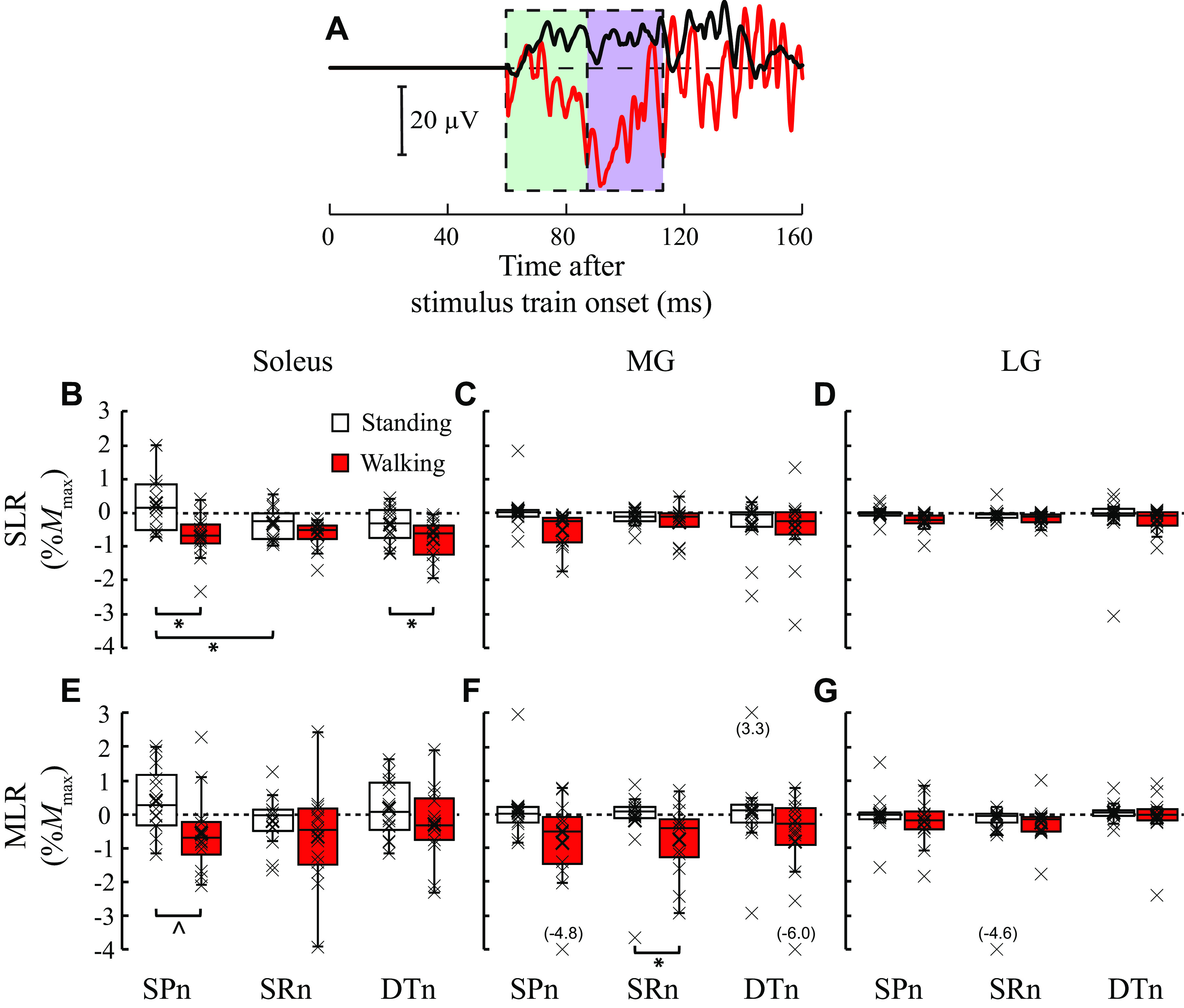
Short latency and medium latency cutaneous reflexes during standing and walking in participants without spinal cord injury (SCI). *A*: examples of soleus [stimulated-nonstimulated (i.e., subtracted)] electromyography (EMG) sweeps after a train of five pulses at 200 Hz of superficial peroneal nerve (SPn) stimulation during standing (black) and during early-to-mid stance phase of walking (red) in a single participant. Responses (10–20) were averaged together for each sweep. Stimulus artifact has been replaced by a flat line from 0 to 60 ms poststimulus. The broken line in the middle indicates the level at which there is no difference between stimulated and nonstimulated EMG; above this line indicates excitation and below indicates inhibition. The green shaded time window is for the short latency response (SLR) and the purple shaded window is for the medium latency response (MLR). *B*–*D*: group data for SLRs [*B* for soleus, *C* for medial gastrocnemius (MG), and *D* for lateral gastrocnemius (LG)] are presented as box-whisker plots with individual data points denoted by *x* (*n* = 14). *E*–*G*: group data for MLRs (*E* for soleus, *F* for MG, and *G* for LG) as box-whisker plots with individuals data points (*x*) (*n* = 14). In *B*–*G*, the reflexes during standing are in white and the reflexes during walking are in red. Significant differences are indicated by * and ^(*P* ≤ 0.05 and 0.001, respectively, by paired *t* test). DTn, distal tibial nerve; SRn, sural nerve.

**Figure 3.**
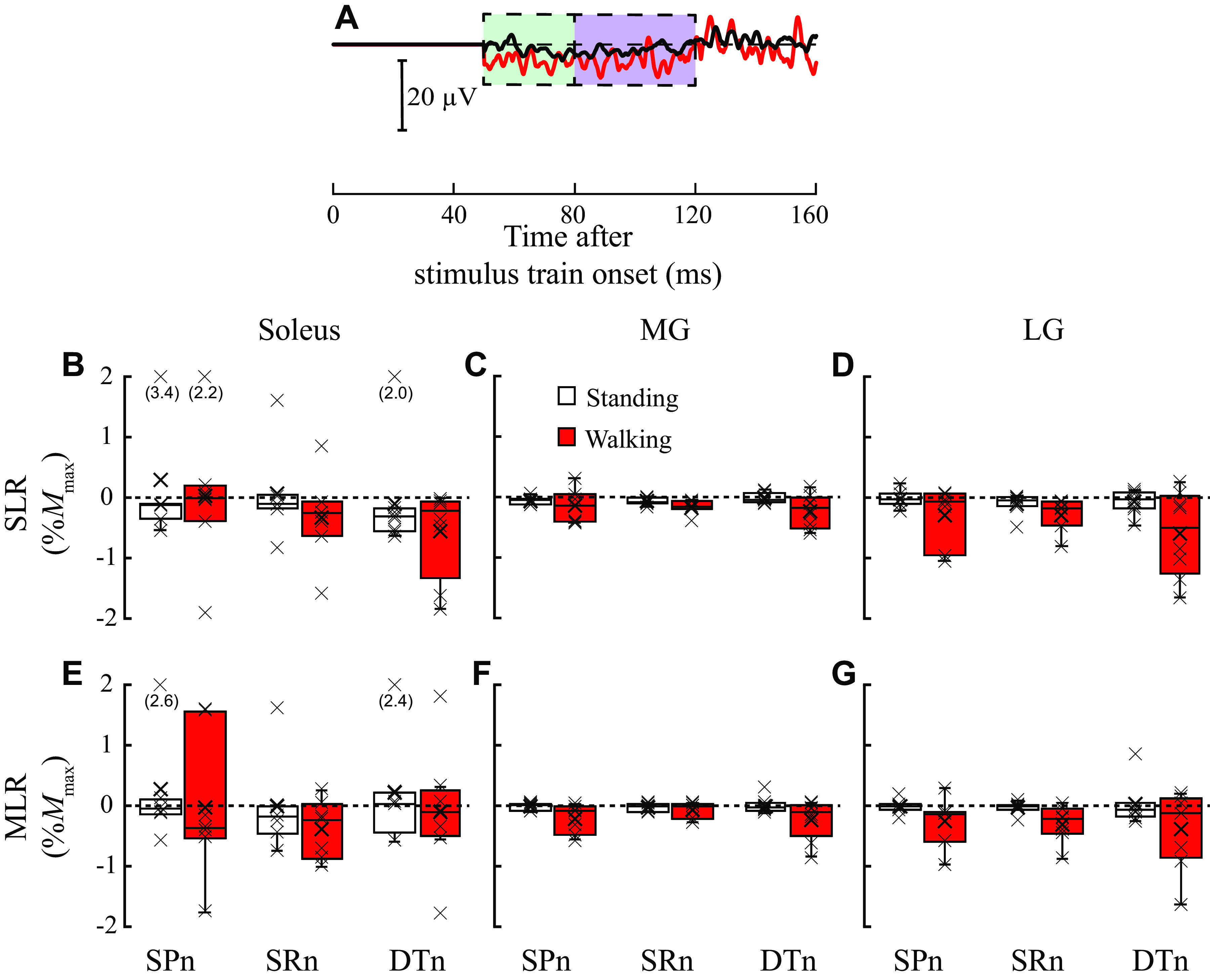
Short and medium latency cutaneous reflexes during standing and walk in participants with spinal cord injury (SCI). *A*: examples of soleus [stimulated-nonstimulated (i.e., subtracted)] electromyography (EMG) sweeps after a train of five pulses at 200 Hz of superficial peroneal nerve (SPn) stimulation during standing (black) and during early-to-mid stance phase of walking (red) in a single participant. Responses (10–20) were averaged together for each sweep. Stimulus artifact has been replaced by a flat line from 0 to 50 ms poststimulus. The broken line in the middle indicates the level at which there is no difference between stimulated and nonstimulated EMG; above this line indicates excitation and below indicates inhibition. The green shaded time window is for the short latency response (SLR) and the purple shaded window is for the medium latency response (MLR). *B*–*D*: group data for SLRs [*B* for soleus, *C* for medial gastrocnemius (MG), and *D* for lateral gastrocnemius (LG)] are presented as box-whisker plots with individual data points denoted by *x* (*n* = 8). *E*–*G*: group data for MLRs (*E* for soleus, *F* for MG, and *G* for LG) as box-whisker plots with individuals data points (*x*) (*n* = 8). In *B–G*, the reflexes during standing are in white and the reflexes during walking are in red. DTn, distal tibial nerve; SRn, sural nerve.

In the non-SCI group, triceps surae SLRs showed both excitation and inhibition, depending on the task and the nerve stimulated. For example, in the soleus SLR, the non-SCI group showed excitation during standing and inhibition during walking with SPn stimulation; and excitation with SPn but inhibition with SRn stimulation during standing. In contrast, in the SCI group, the triceps SLRs were mostly inhibitory across all three nerve stimulation conditions, with clearer suppression of ongoing EMG during walking than during standing. Figure 2*A* and Fig. 3*A* show examples of the soleus EMG sweeps from one participant without SCI (Fig. 2*A*) and one participant with SCI (Fig. 3*A*) following SPn stimulation during walking (red lines) and standing (black lines).

In the non-SCI group, the effects of task (*F*_1,13_ = 9.161, *P* = 0.010, partial η^2^ = 0.413) and nerve (*F*_2,26_ = 3.995, *P* = 0.031, partial η^2^ = 0.235) and task × nerve interaction (*F*_2,26_ = 6. 471, *P* = 0.005, partial η^2^ = 0.332) were significant for the soleus SLR. Post hoc indicated significant differences between standing and walking (i.e., task-dependent modulation) with SPn (0.31%*M*_max_ for standing and −0.68%*M*_max_ for walking, *P* = 0.005) and with DTn (−0.34%*M*_max_ for standing and −0.76%*M*_max_ for walking, *P* = 0.027), and between SPn (0.31%*M*_max_) and SRn (−0.31%*M*_max_) stimulation during standing (*P* = 0.014) (i.e., nerve specificity). There were no main effects of task (*F*_1,13_ = 2.797, *P* = 0.118, partial η^2^ = 0.177), nerve (*F*_2,26_ = 0.895, *P* = 0.421, partial η^2^ = 0.064), or task × nerve interaction (*F*_1.277,16.605_ = 1.389, *P* = 0.265, partial η^2^ = 0.097) in MG SLRs. The effect of task (*F*_1,13_ = 2.675, *P* = 0.126, partial η^2^ = 0.171), nerve (*F*_1.122,14.592_ = 0.386, *P* = 0.568, partial η^2^ = 0.029), or task × nerve interaction (*F*_1.074,13.962_ = 0.249, *P* = 0.642, partial η^2^ = 0.019) was similarly nonsignificant in LG SLR. Cohen’s *D* effect sizes in the non-SCI group for task were small to moderate (0.66, 0.29, and 0.21 in the soleus, MG, and LG, respectively). For nerve, Cohen’s *D* was small for the soleus (0.16–0.45), MG (0.02–0.29), and LG (0.08–0.15). Figure 2, *B*–*D* shows the soleus, MG, and LG SLRs for the non-SCI group.

In individuals with SCI, ANOVA revealed no significant effects of task (soleus *F*_1,6_ = 2.283, *P* = 0.182, partial η^2^ = 0.276; MG *F*_1,6_ = 2.895, *P* = 0.140, partial η^2^ = 0.325; and LG *F*_1,6_ = 3.765, *P* = 0.100, partial η^2^ = 0.386), nerve (soleus *F*_1.074,6.444_ = 1.818, *P* = 0.225, partial η^2^ = 0.233; MG *F*_2,12_ = 0.048, *P* = 0.953, partial η^2^ = 0.008; and LG *F*_1.063,6.379_ = 0.604, *P* = 0.475, partial η^2^ = 0.091), or task × nerve interaction (soleus *F*_2,12_ = 0.156, *P* = 0.857, partial η^2^ = 0.025; MG *F*_2,12_ = 0.129, *P* = 0.880, partial η^2^ = 0.021; and LG *F*_2,12_ = 0.571, *P* = 0.580, partial η^2^ = 0.087) for SLRs. Cohen’s *D* effect sizes for task were moderate in the soleus, MG, and LG (0.51, 0.50, and 0.64, respectively). For the effect of nerve stimulated, effect sizes were small to moderate in the soleus (0.16–0.52) and small in the MG (0.04–0.07) and LG (0.16–0.30). Figure 3, *B*–*D* shows the soleus, MG, and LG SLRs for the SCI group.

### Medium Latency Response during Standing and Walking

Similar to their SLRs, the non-SCI group’s soleus and MG MLRs showed excitation and inhibition depending on the task and nerve stimulated. Overall, the SCI group’s triceps surae MLRs followed a similar pattern as the SLR; that is, MLRs were inhibitory across all three nerve stimulation conditions with somewhat clearer inhibition during walking than during standing.

In the non-SCI group, the effects of nerve and of task × nerve interaction were not significant for the soleus (*F*_1.429,18.579_ = 1.258, *P* = 0.294, partial η^2^ = 0.088; *F*_2,26_ = 1.973, *P* = 0.159, partial η^2^ = 0.132, respectively) whereas task was significant for the soleus MLR (*F*_1,13_ = 10.643, *P* = 0.006, partial η^2^ = 0.450). Post hoc indicated a significant difference between standing and walking (i.e., task-dependent modulation) with SPn (0.43% vs. −0.54%*M*_max_, respectively) (*P* < 0.001). For the MG MLR, effect of nerve and task × nerve interaction were not significant (*F*_2,26_ = 0.094, *P* = 0.910, partial η^2^ = 0.007; *F*_2,26_ = 0.339, *P* = 0.715, partial η^2^ = 0.025, respectively). Effect of task was significant for the MG MLR (*F*_1,13_ = 9.102, *P* = 0.010, partial η^2^ = 0.412). Post hoc indicated a significant difference between standing (−0.17%) and walking (−0.74%) with SRn stimulation (*P* = 0.015) (i.e., task-dependent modulation). There were no main effects of task (*F*_1,13_ = 0.123, *P* = 0.732, partial η^2^ = 0.009), nerve (*F*_2,26_ = 1.744, *P* = 0.195, partial η^2^ = 0.118), or task × nerve interaction (*F*_1.177,15.297_ = 0.732, *P* = 0.427, partial η^2^ = 0.053) found in the LG MLR. Cohen’s *D* effect sizes in the non-SCI group for task were small to moderate in the soleus, MG, and LG (0.64, 0.57, and 0.06, respectively). For the effect of nerve stimulated, effect sizes were small to moderate in the soleus (0.01–0.37) and small in the MG (0.01–0.09) and LG (0.16–0.28). Figure 2, *E*–*G* shows the soleus, MG, and LG MLRs for the non-SCI group.

In individuals with SCI, ANOVA revealed no significant effect of task (soleus *F*_1,6_ = 2.746, *P* = 0.149, partial η^2^ = 0.314; MG *F*_1,6_ = 4.652, *P* = 0.74, partial η^2^ = 0.437; and LG *F*_1,6_ = 4.240, *P* = 0.085, partial η^2^ = 0.414), nerve (soleus *F*_2,12_ = 0.910, *P* = 0.429, partial η^2^ = 0.132; MG *F*_2,12_ = 0.933, *P* = 0.420, partial η^2^ = 0.135; LG *F*_2,12_ = 0.302, *P* = 0.745, partial η^2^ = 0.048), or task × nerve interaction for MLRs (soleus *F*_2,12_ = 0.548, *P* = 0.592, partial η^2^ = 0.084; MG *F*_2,12_ = 2.974, *P* = 0.089, partial η^2^ = 0.331; and LG *F*_2,12_ = 1.017, *P* = 0.391, partial η^2^ = 0.145). Cohen’s *D* effect sizes for task were moderate in the soleus, MG, and LG (0.55, 0.66, and 0.66, respectively). For the effect of nerve stimulated, effect sizes were negligible to small in all muscles (0.02–0.39). Figure 3, *E*–*G* shows the soleus, MG, and LG MLRs for the SCI group.

### H-Reflexes and Their Relation to Cutaneous Reflexes

In all three muscles, the *H*_max_ (in %*M*_max_) was significantly larger for the SCI group than for the non-SCI group: soleus [69 ± 26%*M*_max_ (means ± SD) for the SCI group vs. 45 ± 24%*M*_max_ for the non-SCI group, *P* = 0.037], MG (38 ± 19%*M*_max_ for the SCI group vs. 12 ± 10%*M*_max_ for the non-SCI group, *P* < 0.001) and LG (41 ± 17%*M*_max_ for the SCI group vs. 11 ± 7%*M*_max_ for the non-SCI group, *P* = 0.001).

To assess the potential link between the excitability of the H-reflex pathway and that of cutaneous reflex pathways, Pearson’s *r* was calculated for the *H*_max_ and SLR and for the *H*_max_ and MLR measured in standing across participants. For the H-reflex and SLRs to SPn stimulation, *r* values were 0.014, 0.071, and 0.010 for the soleus (*P* = 0.954), MG (*P* = 0.759), and LG (*P* = 0.966), respectively. *R* values for the H-reflex-SLR correlation with SRn stimulation were 0.367 (*P* = 0.102), 0.238 (*P* = 0.298), and 0.154 (*P* = 0.505), and *r* values for DTn stimulation were 0.160 (*P* = 0.477), 0.315 (*P* = 0.153), and 0.006 (*P* = 0.977), respectively. The findings were similar when r values were calculated for each group separately.

For the H-reflex and MLRs to SPn stimulation, *r* values were 0.063 (*P* = 0.785), 0.103 (*P* = 0.656), and 0.080 (*P* = 0.731) for the soleus, MG, and LG, respectively. With SRn stimulation, they were 0.148 (*P* = 0.523), 0.119 (*P* = 0.606), and 0.032 (*P* = 0.890) and with DTn stimulation they were 0.155 (*P* = 0.492), 0.019 (*P* = 0.934), and 0.222 (*P* = 0.321).

## DISCUSSION

### Altered Modulation of Triceps Surae Cutaneous Reflexes after SCI

This study examined whether cutaneous reflexes are modulated task dependently and with nerve specificity in the triceps surae of people with chronic incomplete SCI. We found that in ambulatory participants with SCI, the reflexes were present in the triceps surae but not modulated significantly between standing and walking; there was no nerve specificity; and the responses were almost always suppressive regardless of the task or nerve stimulated. This contrasts with the observations in the non-SCI (i.e., age-matched control) group of participants who exhibit both excitatory and inhibitory responses depending on the task and nerve stimulated.

Cutaneous reflexes are thought to involve a network of spinal neurons including parallel excitatory and inhibitory interneuronal pathways (37, 51, 52), which would permit the reflex reversal from excitatory to inhibitory (or inhibitory to excitatory) (6, 7) depending on the task (Fig. 2) and/or movement phase (8–13), as in the case for Ib afferent pathways. In plantarflexor MG, reversal of the extensor group Ib afferent action from inhibitory (in nonlocomotion state) to excitatory (in locomotion state) occurs, presumably through the opening of excitatory polysynaptic pathways while suppressing transmission in the disynaptic inhibitory pathways, much like those described in the central pattern generator (CPG) extensor half-center model (53–55). Assuming similar interneuronal pathways existing for cutaneous Aβ afferents, the lack of reflex reversal suggests altered behaviors of those spinal pathways after SCI.

As to the potential origin of altered cutaneous reflex modulation, direct injuries to the interneurons involved in the triceps cutaneous reflexes are unlikely, since all but one of the present participants with SCI had cervical injury, and none lumber SCI. Rather, it would likely be from a problem in modulating the excitability of interneurons associated with cutaneous afferents. Following SCI, the excitability of spinal interneurons below the lesion is generally reduced (56, 57), although motoneuron excitability tends to increase (33, 58, 59), unable to produce meaningful functions. The fact that electrical spinal cord stimulation can increase spinal interneuron excitability by bringing membrane potentials closer to the firing threshold (60, 61) and that epidural (62–64) and transcutaneous (65) spinal cord stimulation can permit the generation of patterned locomotor-like activity support the view that the interneuronal excitability is less modulable when spinal excitability is generally reduced (and thus, when spinal cord stimulation increases the spinal excitability, the interneuronal excitability becomes modulable and starts to produce locomotor-like activity). Furthermore, in the chronic absence of (excitatory) supraspinal input due to SCI, the state of spinal cord pathways could be at an imbalance, chronically stuck in a state that is nonresponsive to volitional or task/function-dependent drive (66, 67). The fact that blocking GABAergic (68) or glycinegic (69) transmission improves locomotion in spinalized animals supports the possibility that modulation of interneuronal excitability is dampened when inhibitory transmission dominates spinal pathways. Thus, it is plausible that limited cutaneous reflex modulation observed in people with SCI reflects their limited ability to modulate spinal interneuronal excitability, as cutaneous reflex behaviors are produced and modulated through a network of interneurons.

### Is the Excitability of the H-Reflex Pathway Related to the Excitability of the Cutaneous Reflex Pathway?

In this study, we found no significant correlation between the excitability of the H- and cutaneous reflex pathways, regardless of injury status. This suggests that the excitability of these two pathways is regulated independently from each other. As to where and how these pathways are regulated separately, in the present study setting, any potential mechanisms that act on motoneurons directly (e.g., excitability of motoneuron pools and inhibitory and excitatory input that converges onto motoneurons including reciprocal and recurrent inhibition) are unlikely ones that separated regulation of these reflexes, since both reflexes were measured while the person was standing and maintaining the background EMG activity. Instead, what could set the reflex gain/behavior in this situation are presynaptic inhibition at the Ia-motoneuron synapses for the H-reflexes, which are often exaggerated in people with chronic SCI (70, 71), and inhibitory or excitatory interneurons that are associated with cutaneous Aβ afferents (51, 72). Thus, the lack of correlation between the H- and cutaneous reflexes may suggest that a group of inhibitory interneurons that produce presynaptic inhibition of Ia-motoneuron pathways and Aβ-associated interneurons do not receive input from the common source or the impact of the common input is outweighed by other input [e.g., possibly Ib and/or joint afferent inputs (73–75)], resulting in producing different reflexes that are not coregulated.

Regardless of the actual neurophysiological mechanisms, the lack of correlation between the reflexes in both injured and uninjured CNS implies the existence of (potentially multiple) parallel and not necessarily cohesive mechanisms to regulate the excitability of spinal pathways. How those different mechanisms and pathways interact with each other in normal and impaired motor control is yet to be understood. Thus, it would be critically important to examine multiple spinal reflexes in individuals with SCI, without injuries, and other neuromuscular disorders, to gain a better understanding of their regulation, modulation, interaction, and plasticity that affect motor control.

### Consideration of Methodological Differences between This and Previous Studies

In interpreting the present findings, the differences in non-SCI participants between this and past studies [i.e., suppressive reflex responses during standing and facilitatory during locomotion (6, 7)] warrant some comments. They could be from differences in reflex windows used for analyses (SLR and MLR in this study vs. cumulative up to 150 ms poststimulus in Ref. 7), muscles recorded from (triceps surae vs. biceps femoris and TA in Ref. 6), and postures (natural standing vs. mimicking late stance or heel strike in Refs. 6 and 7). The stimulus intensity that is known to affect cutaneous reflexes (12) differed; and the participant age that affects somatosensory reflexes (76) differed (48.3 ± 15.0 yr vs. 24–36 yr in Ref. 7). Thus, for interpreting the present cutaneous reflex data in individuals with SCI, we referenced the data from the present age-matched non-SCI participants in whom the experimental and analysis methods were comparable.

As another methodological factor that needs to be mentioned here is a possible impact of walking speed on the appearance of task-dependent modulation. That is, in the present group of individuals with SCI, due to gait impairments, their comfortable walking speeds that were used for locomotor reflex measurements were significantly lower than those of the age-matched non-SCI participants (see Table 1). While if and how walking at a very low speed affected cutaneous reflex modulation are currently unknown, walking at a low speed itself (without reflex elicitation) could affect locomotor EMG pattern and amplitude (77) and thus could be a partial reason for why we did not see clear task-dependent modulation between standing and walking in individuals with SCI in the present study. This will need to be addressed in a future study.

One may wonder about certain specifics of the experimental methods, such as, whether the application of nerve stimulation itself would affect gait, whether functional levels of the studied participants with SCI affected the study findings, and if the present findings are generalizable to a broader population of individuals with SCI. Based on the available studies and data, currently, it is not known whether non-nociceptive cutaneous nerve stimulation alters joint motion in any or all phases of nonstimulated steps. For the stimulated steps, previous studies report that non-nociceptive stimulation that elicited cutaneous reflexes may affect the ankle joint motion to limited extent (i.e., insignificant and <4°) in early-to-mid stance phase (i.e., the phase studied here) but robustly (estimated to be ∼6–9°) in early-to-mid swing phase (8, 16). Little is known about these effects in people after SCI. Thus, in the future study with ambulatory individuals with SCI, we should consider investigating cutaneous reflexes and their effects on joint motion at different phases of the gait cycle. As to the generalizability of study findings and whether participants’ functional levels affected the study findings, the present study does not address them adequately. The present data set is small in size and does not include lower extremity motor screes. Clearly, these aspects ought to be improved in future studies.

It should be noted that the present study set out to investigate whether the mechanisms that regulate the triceps surae cutaneous reflex modulation are functioning normally in individuals with SCI. This scope is different from investigating if and how cutaneous afferent excitation may affect the excitability and behavior of other spinal reflexes; such areas have been investigated by others (e.g., see Refs. 39, 40, and 78–80). It is important to understand how sensory-sensory afferent interaction (25, 74, 81, 82) works in individuals after SCI, as such understanding may help to develop potentially robust neuromodulation rehabilitation strategies in the future.

### Conclusions

This is the first study to examine task-dependent modulation of cutaneous reflexes in individuals with SCI and showed no significant task-dependent or nerve-specific modulation of the triceps surae reflexes in the clinically more spastic leg. Observationally, in the SCI group the reflexes during walking were more suppressive than those during standing, somewhat following the trends in the non-SCI group. This supports the possibility that the pathways involved in cutaneous reflex modulation were present but not fully available in the present group of individuals with chronic incomplete SCI. Presuming that the present findings will be confirmed in future studies with larger sample sizes, the presence, amplitude, and direction (i.e., inhibitory vs. excitatory) of cutaneous reflexes to stimulation of different nerves during different tasks may serve as useful biomarkers for indicating the state of spinal pathways in people after SCI (83, 84).

## DATA AVAILABILITY

Data will be made available upon reasonable request.

## GRANTS

This work was supported in part by South Carolina Spinal Cord Injury Research Fund SCIRF No. 2019 PD-01, 2021 PD-01, NIH National Institute of Neurological Disorders and Stroke (NINDS) R01 NS114279 (to A. K. Thompson), NIH National Center of Neuromodulation for Rehabilitation (NICHD) P2C HD086844 (to S. A. Kautz), National Institute of Biomedical Imaging and Bioengineering (NIBIB) P41EB018783 (to J. R. Wolpaw), the Doscher Neurorehabilitation Research Program, and New York State Spinal Cord Injury Research Trust C33279GG (to J. R. Wolpaw).

## DISCLOSURES

No conflicts of interest, financial or otherwise, are declared by the authors.

## AUTHOR CONTRIBUTIONS

A.M.P. and A.K.T. conceived and designed research; A.M.P. and A.K.T. performed experiments; A.M.P. and A.K.T. analyzed data; A.M.P. and A.K.T. interpreted results of experiments; A.M.P. prepared figures; A.M.P. drafted manuscript; A.M.P. and A.K.T. edited and revised manuscript; A.M.P. and A.K.T. approved final version of manuscript.
